# Supplementary material for: Multi‐dataset identification of innovative feature genes and molecular mechanisms in keratoconus
Source: J Cell Mol Med. 2024 Sep 19;28(18):e70079. doi: 10.1111/jcmm.70079 (PMC11412914; doi:10.1111/jcmm.70079)
Supplement: Supplementary file 2 — Material S2. [file JCMM-28-e70079-s002.zip › 7.GSEA analysis/4.MYRF/KEGG_VIRAL_MYOCARDITIS.html]

Details for gene set KEGG\_VIRAL\_MYOCARDITIS[GSEA]

|  || Dataset | MYRF.group.cls#h\_versus\_l |
| Phenotype | group.cls#h\_versus\_l |
| Upregulated in class | l |
| GeneSet | KEGG\_VIRAL\_MYOCARDITIS |
| Enrichment Score (ES) | -0.43753102 |
| Normalized Enrichment Score (NES) | -1.3873821 |
| Nominal p-value | 0.11885246 |
| FDR q-value | 0.24997671 |
| FWER p-Value | 0.894 |
Table: GSEA Results Summary

  

Fig 1: Enrichment plot: KEGG\_VIRAL\_MYOCARDITIS      
 Profile of the Running ES Score & Positions of GeneSet Members on the Rank Ordered List

  

| SYMBOL | TITLE | RANK IN GENE LIST | RANK METRIC SCORE | RUNNING ES | CORE ENRICHMENT || 1 | CD86 | na | 355 | 0.626 | 0.0421 | No |
| 2 | RAC3 | na | 2468 | 0.325 | 0.0083 | No |
| 3 | ITGAL | na | 2764 | 0.308 | 0.0255 | No |
| 4 | MYH11 | na | 4047 | 0.252 | 0.0096 | No |
| 5 | DAG1 | na | 4906 | 0.225 | 0.0037 | No |
| 6 | MYH15 | na | 5198 | 0.216 | 0.0134 | No |
| 7 | HLA-DOA | na | 8167 | 0.134 | -0.0610 | No |
| 8 | EIF4G3 | na | 8455 | 0.128 | -0.0586 | No |
| 9 | CASP3 | na | 8824 | 0.120 | -0.0592 | No |
| 10 | CASP8 | na | 9334 | 0.109 | -0.0648 | No |
| 11 | MYH14 | na | 10451 | 0.087 | -0.0896 | No |
| 12 | MYH13 | na | 10964 | 0.079 | -0.0978 | No |
| 13 | MYH6 | na | 11121 | 0.076 | -0.0960 | No |
| 14 | CD28 | na | 12283 | 0.059 | -0.1245 | No |
| 15 | ACTB | na | 12485 | 0.057 | -0.1256 | No |
| 16 | MYH7 | na | 12644 | 0.055 | -0.1255 | No |
| 17 | HLA-B | na | 12962 | 0.051 | -0.1305 | No |
| 18 | BID | na | 13268 | 0.047 | -0.1354 | No |
| 19 | DMD | na | 14406 | 0.033 | -0.1654 | No |
| 20 | MYH8 | na | 14456 | 0.033 | -0.1640 | No |
| 21 | CCND1 | na | 14657 | 0.031 | -0.1672 | No |
| 22 | MYH2 | na | 15413 | 0.024 | -0.1870 | No |
| 23 | HLA-F | na | 16261 | 0.015 | -0.2102 | No |
| 24 | HLA-E | na | 16263 | 0.015 | -0.2090 | No |
| 25 | RAC2 | na | 17259 | 0.006 | -0.2372 | No |
| 26 | MYH10 | na | 17326 | 0.006 | -0.2386 | No |
| 27 | HLA-C | na | 17654 | 0.003 | -0.2478 | No |
| 28 | CD55 | na | 18969 | -0.010 | -0.2849 | No |
| 29 | EIF4G2 | na | 19046 | -0.011 | -0.2861 | No |
| 30 | HLA-A | na | 20084 | -0.023 | -0.3142 | No |
| 31 | HLA-DRB5 | na | 20183 | -0.024 | -0.3150 | No |
| 32 | HLA-G | na | 20210 | -0.024 | -0.3137 | No |
| 33 | MYH9 | na | 20365 | -0.026 | -0.3160 | No |
| 34 | EIF4G1 | na | 21730 | -0.042 | -0.3518 | No |
| 35 | ITGB2 | na | 21824 | -0.044 | -0.3508 | No |
| 36 | RAC1 | na | 21936 | -0.045 | -0.3503 | No |
| 37 | CXADR | na | 22294 | -0.049 | -0.3564 | No |
| 38 | ACTG1 | na | 22317 | -0.050 | -0.3529 | No |
| 39 | CASP9 | na | 23052 | -0.060 | -0.3690 | No |
| 40 | HLA-DRB3 | na | 23156 | -0.062 | -0.3668 | No |
| 41 | LAMA2 | na | 23159 | -0.062 | -0.3617 | No |
| 42 | CYCS | na | 23572 | -0.069 | -0.3678 | No |
| 43 | ABL1 | na | 23912 | -0.074 | -0.3714 | No |
| 44 | HLA-DQB1 | na | 24038 | -0.076 | -0.3686 | No |
| 45 | HLA-DRB1 | na | 24046 | -0.077 | -0.3624 | No |
| 46 | HLA-DOB | na | 24105 | -0.078 | -0.3576 | No |
| 47 | MYH3 | na | 25603 | -0.105 | -0.3921 | No |
| 48 | CD80 | na | 26452 | -0.122 | -0.4063 | No |
| 49 | HLA-DMA | na | 27498 | -0.148 | -0.4241 | Yes |
| 50 | SGCA | na | 27650 | -0.152 | -0.4158 | Yes |
| 51 | MYH1 | na | 27858 | -0.157 | -0.4087 | Yes |
| 52 | SGCG | na | 28859 | -0.187 | -0.4219 | Yes |
| 53 | SGCD | na | 29214 | -0.197 | -0.4156 | Yes |
| 54 | MYH4 | na | 29629 | -0.211 | -0.4100 | Yes |
| 55 | HLA-DRB4 | na | 30247 | -0.232 | -0.4084 | Yes |
| 56 | ABL2 | na | 30308 | -0.234 | -0.3905 | Yes |
| 57 | CD40LG | na | 30708 | -0.251 | -0.3811 | Yes |
| 58 | PRF1 | na | 31281 | -0.279 | -0.3743 | Yes |
| 59 | HLA-DPA1 | na | 32053 | -0.320 | -0.3697 | Yes |
| 60 | SGCB | na | 32475 | -0.349 | -0.3528 | Yes |
| 61 | CD40 | na | 32572 | -0.356 | -0.3258 | Yes |
| 62 | MYH7B | na | 32705 | -0.366 | -0.2990 | Yes |
| 63 | HLA-DRA | na | 33031 | -0.390 | -0.2758 | Yes |
| 64 | HLA-DMB | na | 33256 | -0.414 | -0.2477 | Yes |
| 65 | CAV1 | na | 33722 | -0.474 | -0.2215 | Yes |
| 66 | FYN | na | 33971 | -0.517 | -0.1855 | Yes |
| 67 | ICAM1 | na | 34123 | -0.548 | -0.1440 | Yes |
| 68 | HLA-DQA2 | na | 34217 | -0.576 | -0.0985 | Yes |
| 69 | HLA-DPB1 | na | 34331 | -0.609 | -0.0509 | Yes |
| 70 | HLA-DQA1 | na | 34602 | -0.745 | 0.0036 | Yes |
Table: GSEA details [plain text format]

  

Fig 2: KEGG\_VIRAL\_MYOCARDITIS      
 Blue-Pink O' Gram in the Space of the Analyzed GeneSet

  

Fig 3: KEGG\_VIRAL\_MYOCARDITIS: Random ES distribution      
 Gene set null distribution of ES for **KEGG\_VIRAL\_MYOCARDITIS**
